# Characterization of the complete chloroplast genome of the traditional medicinal plants *Rhodiola rosea* (Saxifragales: Crassulaceae)

**DOI:** 10.1080/23802359.2018.1483774

**Published:** 2018-07-10

**Authors:** Dan-Ni Zhao, Jian-Qiang Zhang

**Affiliations:** College of Life Sciences, Shaanxi Normal University, Xi’an, China

**Keywords:** *Rhodiola rosea*, chloroplast genome, phylogeny

## Abstract

*Rhodiola rosea* L. is used in herbal medicine in many countries for a long time. Here, its complete chloroplast genome was assembled and annotated. The genome is 151,348 bp long and comprises a pair of inverted repeat regions (IRs, 25,790 bp each), a large single-copy region (LSC, 82,716 bp), and a small single-copy region (SSC, 17,052 bp). It contained 113 gene species (79 protein coding, 29 tRNA, 4 rRNA, and 1 pseudogene), with 20 of them occurring in double copies. Introns were detected in 12 PCG and 5 tRNA species. The nucleotide composition is inhomogeneous (30.9% A, 19.2% C, 18.5% G, and 31.4% T) with an overall A + T content of 62.3%. Phylogenetic analysis indicated that *Rhodiola rosea* is sister to the remaining species of *Rhodiola* with maximum support in phylogeny.

Plants have been important footstones of a complex traditional medical system that has produced some of the most important drugs that still exist today (Gurib-Fakim 2006). *Rhodiola rosea* is a species of herbaceous perennials within the family Crassulaceae (Fu and Ohba 2001), whose roots and rhizomes are famous medicinal materials in many countries (Zhu et al. 2017). Here, we assembled its complete chloroplast (cp) genome and investigated its phylogenetic placement within Crassulaceae, to make use of *R. rosea* more efficiently (Famà et al. 2002; Mukherjee et al. 2016).

Using the CTAB method, total genomic DNA was isolated from silica-gel dried leaves of an individual of *R. rosea* from Mt. Xiaowutai (Hebei Province, China; N 39° 57′ 20″, E 115° 04′ 08″'). The voucher specimen was stored in the Peking University Herbarium (PEY) with accession number J. Q. Zhang et al. 120613-01. With *R. ovatisepala* as the starting reference, the clean reads were used to assemble the cp genome with the program MITObim v1.9 (Hahn et al. 2013) after 29.8 M of 150-bp raw paired reads were trimmed using CLC Genomics Workbench v8 (CLC Bio, Aarhus, Denmark). The genome was annotated with Geneious R10 (Biomatters Ltd., Auckland, New Zealand) using the cp genome of *R. ovatisepala* as the reference.

The cp genome of *R. rosea* is 151,348 bp long exhibiting a typical quadripartite structure. The large (LSC, 82,716 bp) and small (SSC, 17,052 bp) single-copy regions were separated by a pair of inverted repeat regions (IRs, 25,790 bp each). It contained 113 gene species (79 protein coding, 29 tRNA and 4 rRNA, and 1 pseudogene). About 20 gene species duplicated, including 9 PCG species (*ndhB, rpl2, rpl23, rps7, rps12, rps19, ycf1*, and *ycf2*), 7 tRNA species (*trnA-UGC, trnI-CAU, trnI-GAU, trnL-CAA, trnN-GUU*, *trnR-ACG*, and *trnV-GAC*), 4 rRNA species (*4.5S, 5S, 16S*, and *23S rRNA*) and one pseudogene (*ycf15*). Of these 20 gene species, three species (*ycf1, rps12,* and *rps19*) partially within the IR regions, while the others completely within the IR regions. Fourteen gene species (*atpF, ndhA, ndhB, petB, petD, rpl2, rpl16, rpoC1, rps16, trnA-UGC, trnI-GAU, trnK-UUU, trnL-UAA,* and *trnV-UAC*) contain one intron, three species (*clpP, rps12* & *ycf3*) possess two introns. The nucleotide composition is asymmetric (30.9% A, 19.2% C, 18.5% G and 31.4% T) with an overall A + T content of 62.3%. The A + T content of the LSC (64.3%) and SSC (68.3%) regions are distinctly higher than those of the IR regions (57.1%).

The most parsimony tree and Bayesian tree were conducted using PAUP* 4.0b10 (Swofford 2003) and MrBayes 3.2.1 (Ronquist and Huelsenbeck 2003), respectively, for 10 species. Three species of *Sedum* are obtained from GenBank (NC023085, NC026065, NC027837) and remaining species were from our own data. Outgroup was chosen as *Adromischus cristatus*. The ingroup can be subdivided into two clades with maximum support (Figure 1). One clade comprised *R. ovatisepala*, *R. humilis*, *R. smithii*, *R. rosea*, and *Sedum takesimense*, and *R. rosea* is sister to the remaining species of *Rhodiola*; the other clade comprised *Rosularia alpestris*, *Dudleya farinose*, *Sedum oryzifolium*, and *Sedum sarmentosum*.

**Figure 1.**
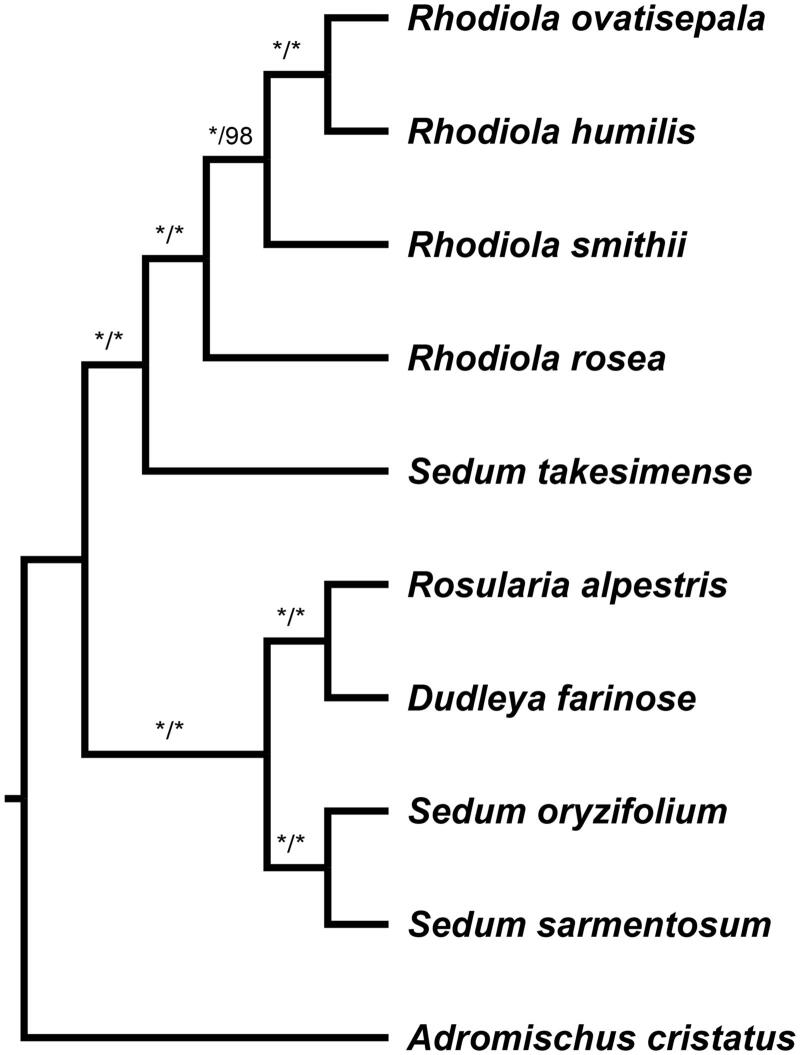
Bayesian tree of 10 Crassulaceae species based on the complete chloroplast genome. Bayesian posterior probabilities (PP) and MP bootstrap (BP) values are shown above the branches (PP/BP). “*” means fully support (1.00/100%).
